# High 15-F_2t_-Isoprostane Levels in Patients with a Previous History of Nonmelanoma Skin Cancer: The Effects of Supplementary Antioxidant Therapy

**DOI:** 10.1155/2015/963569

**Published:** 2015-10-05

**Authors:** Betânia de Jesus e Silva de Almendra Freitas, Gustavo Rafaini Lloret, Marília Berlofa Visacri, Bruna Taliani Tuan, Lais Sampaio Amaral, Daniele Baldini, Vanessa Marcílio de Sousa, Laís Lima de Castro, Jordana Rayane Sousa Aguiar, Eder de Carvalho Pincinato, Priscila Gava Mazzola, Patricia Moriel

**Affiliations:** ^1^Department of Nutrition, Federal University of Piauí (UFPI), Campus Universitário Ministro Petrônio Portella, 64049-550 Teresina, PI, Brazil; ^2^School of Medical Sciences (FCM), University of Campinas (UNICAMP), 13083-887 Campinas, SP, Brazil; ^3^Faculty of Pharmaceutical Sciences (FCF), University of Campinas (UNICAMP), 13083-859 Campinas, SP, Brazil; ^4^Department of Biological and Health Science Center, Mackenzie Presbyterian University, 01302-907 São Paulo, SP, Brazil

## Abstract

*Background*. Phase I of this study was aimed at comparing the profiles of oxidative stress biomarkers in patients with history of nonmelanoma skin cancer (NMSC), previously treated with surgery, to the healthy subjects. Phase II aimed to evaluate the effects of supplementary antioxidant therapy on the levels of biomarkers in the case group. *Materials and Methods*. In Phase I, oxidative stress biomarkers were measured in blood samples obtained from 24 healthy subjects and 60 patients with history of NMSC previously treated with surgery. In Phase II, the 60 patients with history of NMSC were randomized into two subgroups, one receiving placebo (*n* = 34) and the other (*n* = 26) receiving vitamin C, vitamin E, and zinc supplementation for 8 weeks, followed by reevaluation of biomarkers. *Results*. In Phase I, patients with history of NMSC showed increased plasma concentrations of all biomarkers, but only 15-F_2t_-isoprostane was significantly higher than in the healthy subjects. Risk of NMSC increased by 4% for each additional 1 pg/mL increase in 15-F_2t_-isoprostane. In Phase II, supplementation did not significantly reduce levels of oxidative stress biomarkers. *Conclusion*. Patients with history of NMSC had significantly high 15-F_2t_-isoprostane plasma levels; supplementation did not result in significant reduction of oxidative stress biomarkers. This trial was registered with ClinicalTrials.gov (ID NCT02248584).

## 1. Introduction

Oxidative stress occurs when there is disequilibrium between the generation of reactive oxygen and nitrogen species (ROS-RNS) and the cellular antioxidant system and may result in significant damage to cellular structures as well as nucleotides, proteins, and lipids. Lipid peroxidation generates a variety of relatively stable decomposition products, such as isoprostanes [1], which can be measured in plasma and urine as indirect biomarkers of cellular prooxidant status.

Substantial evidence suggests that oxidative stress is a prominent feature of many acute and chronic diseases including cancer, cardiovascular disease, and neurodegenerative pathologies, as well as being part of the normal aging process [2, 3]. Oxidant-mediated alterations may contribute to numerous skin disorders ranging from photosensitivity to cancer. This has led to the search for nontoxic antioxidants that could potentially minimize and/or reverse these changes [4].

Skin cancer is one of the most important and common cancers, with considerably more than a million new cases diagnosed each year worldwide [5]. Skin cancer is commonly grouped into two different categories, melanoma skin cancer (MSC) and nonmelanoma skin cancer (NMSC), based on the cell of origin and clinical behavior. The incidence of NMSC is much higher than that of melanoma. Fortunately, the vast majority of NMSCs are much easier to treat than MSCs and have a considerably better long-term prognosis. There are strong epidemiologic and molecular data linking all forms of skin cancer to sunlight exposure, and it is estimated that ultraviolet (UV) radiation is the primary cause of nearly 90% of NMSCs [6]. A component of sunlight is UVA radiation, which penetrates deeper into the skin than UVB due to its longer wavelengths. While UVA can indirectly damage DNA through the formation of ROS, such as the superoxide anion, hydrogen peroxide, and the hydroxyl radical [7], UVB can directly damage DNA leading to the apoptosis of sunburn cells in skin [5]. ROS may induce extensive DNA damage, lead to cross-links between DNA and proteins, and cause DNA and chromosomal aberrations that may be mutagenic [8]. ROS may also be involved in tumorigenesis through the activation of procarcinogens to generate free radicals that can attack nucleophiles [4].

The purpose of the present study was to compare the profiles of oxidative stress biomarkers, especially 15-F_2t_-isoprostane, of patients with a history of NMSC previously treated by surgery (case group) with those of healthy subjects (comparison group) (Phase I) and to determine whether supplementation with antioxidants could influence the levels of biomarkers in the case group (Phase II).

## 2. Materials and Methods

This was a two-phase study composed of Phase I, with a cross-sectional study design, and Phase II, a double-blind, randomized, placebo-controlled trial, carried out between January 2011 and December 2011 in Teresina, Piauí, Brazil. The study was approved by the local ethics committee, and all participants provided a signed informed consent form authorizing use of their data.

### 2.1. Characterization of Groups, Recruitment, and Eligibility

In Phase I, the case group (*n* = 64) consisted of patients with a history of NMSC (squamous cell carcinoma or basal cell carcinoma) treated with surgery, recruited from a hospital in Teresina, Piauí, while the comparison group (*n* = 24) was composed of workers from a university in Teresina, Piauí, with no history of NMSC. In Phase II, the patients in the case group were randomized into two subgroups, one receiving placebo (*n* = 34) and the other receiving supplementary antioxidant therapy (*n* = 26).

The inclusion criteria for all participants were as follows: age ≥20 years; absence of comorbidities such as type 1 diabetes, severe heart disease, hepatic dysfunction, renal failure requiring dialysis, HIV infection, or MSC; no history of chemotherapy or radiotherapy in the previous 6 months; absence of severe psychiatric disease that limited comprehension; and no taking of any vitamin and/or mineral supplementation. Subjects in the case group who did not complete the entire course of supplemental therapy were excluded from the study during Phase II.

### 2.2. Sociodemographic Characteristics

Sociodemographic characteristics of both the comparison and case groups were collected using a standardized form that included age, gender, race, smoking and alcohol habits (yes or no), actual use of sunscreen, sunlight exposure, and family history of cancer. In the case group, information regarding histopathological parameters and tumor localization was also collected.

### 2.3. Anthropometric and Dietary Investigations

Anthropometric characteristics were assessed for both groups, and a food frequency questionnaire was used to estimate the daily intake of vitamins and minerals for each patient. Dietary reference intakes (DRIs) established by the Institute of Medicine of the National Academies were used to evaluate the necessity of supplemental therapy (Table 1).

### 2.4. Supplemental Antioxidant Therapy

The placebo group and supplemented group received, respectively, a placebo capsule containing only lactose and an identical capsule containing vitamin C (500 mg; CVS Quality, USA), vitamin E (400 IU; CVS Quality), and zinc (50 mg; Nature's Bounty, USA) per day for 60 days. The concentrations of antioxidants administered were determined according to the DRI Tolerable Upper Intake Level (UL) and recommendations of the Institute of Medicine/National Academy Press [9, 10] (Table 1).

### 2.5. Oxidative Stress Biomarkers

Oxidative stress biomarkers including 15-F_2t_-isoprostane, thiobarbituric acid reactive substances (TBARS), nitrite, and total antioxidant capacity (TAC) were measured in plasma. All analyses were performed for both the comparison and case groups. The comparison group was evaluated only in Phase I. The case group was evaluated at two different times: (a) one day before the start of supplementation or placebo administration and (b) one day after the finish of intervention period. A total of 10 mL of venous blood was obtained from all subjects. Blood was withdrawn into blood collection tubes with EDTA or citrate as an anticoagulant and centrifuged (2500 rpm for 10 min at 4°C), and the plasma was stored at −80°C until analysis. Each test for oxidative stress biomarkers required 1 mL plasma. The plasma concentration of 15-F_2t_-isoprostane was determined by enzyme-linked immunosorbent assay (Isoprostane Express EIA Kit; Cayman, USA). Plasma TBARS was measured using a colorimetric method as previously described [11], and the values were expressed in nmol per mL of malondialdehyde equivalents. TAC was assessed using an Antioxidant Assay Kit (Cayman, USA). Nitric oxide synthesis was assessed through nitrite measurement using the Griess assay [12] expressed in *μ*mol of nitrite.

### 2.6. Data Analysis

Descriptive analyses were performed on absolute frequencies and percentages for categorical variables. Numerical variables were described by the mean and standard deviation. Statistical analyses were performed using the SAS System for Windows 9.3 program and data were analyzed using Chi-square, Fisher, Mann-Whitney, or ANOVA for repeated measures tests. Multivariate logistic regression analysis was performed to identify risk factors for NMSC. The following covariates were used: age, race, smoking, alcoholism, family history of cancer, 15-F_2t_-isoprostane, TBARS, nitrite, and TAC, and two-sided *p* values < 0.05 were considered statistically significant. When the sociodemographic characteristics differed between the groups, the data were normalized and used in all regression analyses.

## 3. Results

### 3.1. Phase I

Sociodemographic characteristics, anthropometric parameters, and levels of daily vitamin intake for both groups (comparison and case groups) are detailed in Table 2. Both groups contained more women than men. Age, prolonged daily sunlight exposure, sunscreen use, and previous family history of skin cancer were all significantly higher in the case group.

Of the patients in the case group (*n* = 60), 85.0% had basal cell carcinoma, while the remaining 15.0% had squamous cell carcinoma. The face (80.0%) was the most common area affected, followed by the trunk (8.3%), neck (6.7%), and other areas (5.0%).

The case group had higher plasma concentrations of all oxidative biomarkers assessed (Figure 2(a)). However, only the increase in 15-F_2t_-isoprostane levels was statistically significant (81.9 ± 36.4 pg/mL versus 46.2 ± 21.8 pg/mL, *p* < 0.0001). Therefore, patients with a previous history of NMSC were considered to have a relatively prooxidant cellular state compared with healthy subjects.

Multivariate logistic regression analysis identified age (*p* = 0.0050, odds ratio (OR) = 1.121, and 95% confidence interval (CI) = 1.035–1.213) and the oxidative stress biomarker 15-F_2t_-isoprostane (*p* = 0.0099, OR = 1.044, and 95% CI = 1.010–1.078) as risk factors associated with NMSC. The risk of NMSC increased by 12% each year and by 4% for each additional 1 pg/mL increase in 15-F_2t_-isoprostane levels in the plasma.

Table 2 shows that levels of daily vitamin C, vitamin E, and zinc intake in the case group were lower than recommended for both genders; therefore, vitamin C, vitamin E, and zinc supplementation was clinically indicated (Phase II).

### 3.2. Phase II

**Figure 1:**
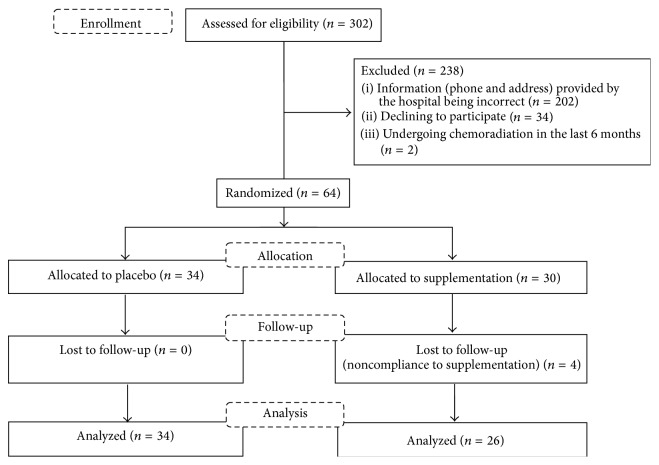
Flow diagram of the passage of participants (Phase II).

The recruitment of participants to Phase II is described in Figure 1. Sociodemographic characteristics, anthropometric parameters, and levels of daily vitamin intake for both groups (placebo and supplemented) are detailed in Table 3. Placebo and supplementation subgroups had similar sociodemographic characteristics, except for alcoholism, which was significantly more prominent in the supplemented subgroup.

**Figure 2:**
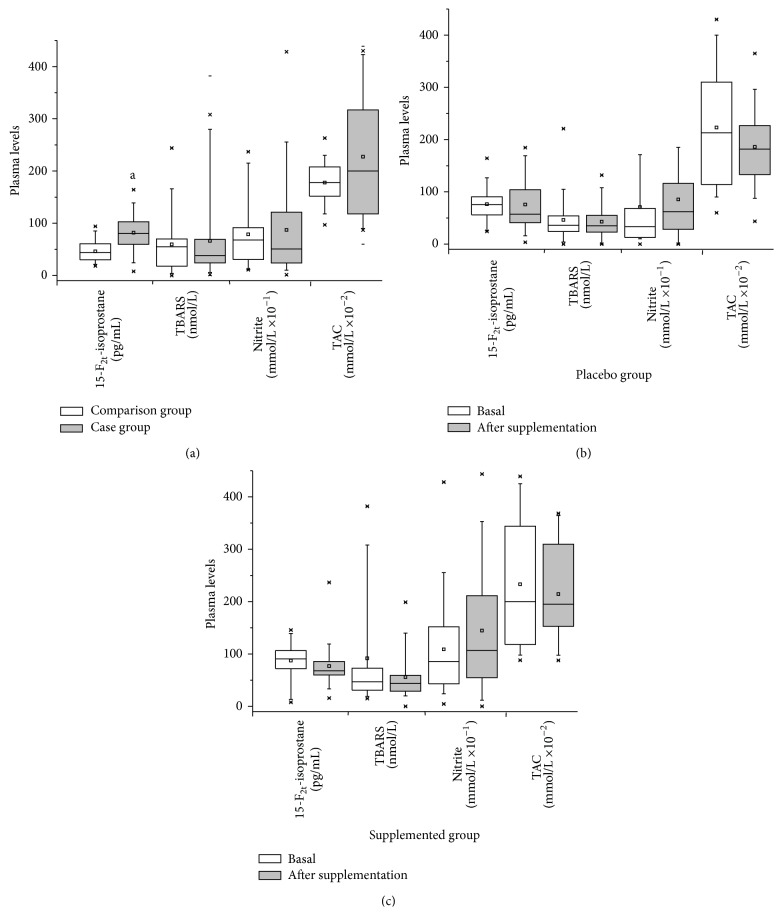
Mean values of oxidative stress biomarkers from each group present in the study: (a) Phase I/comparison and case groups, (b) Phase II/case group (placebo): basal and after supplementation, and (c) Phase II/case group (supplemented): basal and after supplementation. ANOVA for repeated measures statistical test; ^a^ statistically significant difference in relation to comparison group.

A reduction of TAC (2.3 mmol/L versus 2.1 mmol/L), 15-F_2t_-isoprostane (87.3 pg/mL versus 76.8 pg/mL), and TBARS (90.0 nmol/L versus 60.0 nmol/L) levels after supplementation with antioxidants was observed, although these differences did not reach statistical significance compared with placebo. Compared with baseline values, the group receiving supplementation showed a reduction of both mean and median plasma levels of 15-F_2t_-isoprostane, in addition to a more homogeneous distribution of values, which was not observed in the placebo group. The supplementation group also showed a small increase of nitrite levels (10.9 mmol/L versus 15.0 mmol/L), but this was not significantly different from that of the placebo group (Figures 2(b) and 2(c)).

## 4. Discussion

Owing to the increasing incidence, morbidity, and mortality of cancer worldwide, new diagnostic and screening methods are needed for early detection. In recent years, knowledge of cancer biomarkers has increased greatly and this presents numerous opportunities to improve cancer management [13]. The present study demonstrated that plasma levels of 15-F_2t_-isoprostane were significantly higher in patients with a previous history of NMSC compared to healthy subjects. Moreover, each additional 1 pg/mL of 15-F_2t_-isoprostane in plasma was associated with a 4% greater chance of developing NMSC. Age was also found to be a risk factor for NMSC. In addition, patients with history of NMSC had a cellular prooxidant state compared to healthy subjects.

Oxidative stress has been implicated in various pathological conditions, including cancer. When evaluating a marker for its importance* in vivo*, difficulties may arise because of analytical problems relating to its specificity and sensitivity [14]. Isoprostanes are certainly the most specific markers of lipid peroxidation but are also the most difficult to measure [14]. Several comprehensive reviews providing information on the biochemistry of isoprostanes and their utilization as markers of oxidative stress have been published in the last decade [2, 14, 15].

Isoprostanes are prostaglandin isomers produced by peroxidation of polyunsaturated fatty acids from the cellular membrane. The most frequent isomer released into the circulation is 15-F_2t_-isoprostane, which has been identified as a promising key biomarker to investigate the role of oxidative injury [16] in several diseases, including cancer. Barocas et al. [17] reported that increased levels of isoprostanes are present in the urine of patients with prostate cancer. Moreover, Rossner Jr. et al. [18] presented evidence that increased levels of isoprostanes may be correlated with an increased risk of breast cancer. Belli et al. [19] showed that 15-F_2t_-isoprostane levels were increased in basal cell carcinoma and in healthy skin previously exposed to UVA irradiation. Increased levels of isoprostanes have also been reported in several acute and chronic diseases including cardiovascular and neurodegenerative pathologies and cancer, as well as during the normal aging process [16, 20].

As oxidative stress is widely believed to cause or aggravate several human pathologies as described above, it is possible that antioxidants could counteract the harmful effects caused by a prooxidant profile and prevent or treat oxidative stress-related diseases. Herein, it was observed that daily intake levels of vitamins C and E in the case group were lower than those recommended (DRI guidelines). After supplementation, there was no significant reduction in levels of oxidative stress biomarkers. However, there was a small reduction in plasma levels of all oxidative biomarkers evaluated, especially 15-F_2t_-isoprostane. Both mean and median plasma levels of 15-F_2t_-isoprostane were reduced after supplementation, and there was a more homogeneous distribution of values in this group. Additional studies on the modulation of isoprostanes by antioxidant nutrients have recently been published. For example, Reilly et al. [21] reported that vitamin C administered to heavy smokers for 5 days at 2 g/day significantly reduced isoprostane excretion in their urine. Moreover, Davi et al. [22, 23] described a significant reduction in urinary isoprostane excretion in patients with diabetes mellitus and hypercholesterolemia after supplemental therapy with vitamin E.

Our study did not show a significant reduction in the levels of 15-F_2t_-isoprostane after supplementation with vitamin C, vitamin E, and zinc for 60 days. However a study conducted by Dietrich et al. [24], in which patients were supplemented for 60 days with vitamin C at 500 mg/day and vitamin E at 300 mg/day, showed a statistically significant reduction in the plasma levels of 15-F_2t_-isoprostane. These doses were similar to those used by our study. In a study using doses of vitamin C lower than 500 mg/day and doses of vitamin E lower than 300 mg/day, a longer period of supplementation did not lead to showing a significant reduction in plasma levels of biomarkers of oxidative stress [25]. In a study by Murer et al. [26], there was a significant reduction of over 30% (*p* = 0.014) of 15-F_2t_-isoprostane levels in the urine of obese children after supplementation with 500 mg/day of vitamin C, 400 IU/day of vitamin E, and 50 mg/day of selenium for 119 days.

In a recent study by Roberts II et al. [27], the influence of the dose of vitamin E on the reduction of 15-F_2t_-isoprostane was examined in patients with coronary artery disease. The study was initially carried out with patients supplemented with 3200 IU/day vitamin E for 20 weeks and then participants were supplemented with a dose of 0, 100, 200, 400, 800, 1600, or 3200 IU/day vitamin E for 16 weeks. Maximum suppression of plasma levels of 15-F_2t_-isoprostane did not occur until the 16th week of supplementation and the percentage reduction in plasma concentrations of 15-F_2t_-isoprostane only reached significance at doses of 1600 IU/day and 3200 IU/day. These data reveal the dose-dependent effects of antioxidant supplementation for the reduction of biomarkers of oxidative stress and indicate that the dose and time of supplementation should be chosen carefully to achieve the desired result.

This study has some limitations: a relatively small sample size; a short period of antioxidant supplementation; a lack of information about cancer types (primarily skin cancer) in families; a lack of past information about smoking, alcohol habits, use of sunscreen, and sunlight exposure; and a lack of a robust classification for smoking, alcohol habits, and use of sunscreen. In addition, the results of the immunoassays could be confounded by the structural similarities between isoprostanes and some prostaglandins; therefore, 15-F_2t_-isoprostane should preferably be quantified by mass spectrometry.

## 5. Conclusion

The present study demonstrated that patients with a history of NMSC had a statistically significant increase in plasma levels of 15-F_2t_-isoprostane. This suggests that oxidative stress may play an important role in the pathogenesis of skin cancers. Moreover, to the authors' knowledge, this is the first demonstration that increased plasma levels of 15-F_2t_-isoprostane are a risk factor for NMSC. The supplementary antioxidant therapy did not cause significant reduction in levels of oxidative stress biomarkers, so there is need for further evaluation of these supplements as long-term adjunctive therapy.

## Figures and Tables

**Table 1:** Dietary reference intakes (UL–DRIs) and recommendations of the Institute of Medicine/National Academy Press [9, 10].

	Gender	DRI
Vitamin A	Male	625 μg/d
	Female	500 μg/d

Vitamin C	Male	75 mg/d
	Female	60 mg/d

Vitamin E	Male	12 mg/d
	Female	12 mg/d

Cuprum	Male	700 μg/d
	Female	700 μg/d

Zinc	Male	9.4 mg/d
	Female	6.8 mg/d

Selenium	Male	45 μg/d
	Female	45 μg/d

**Table 2:** Comparison regarding sociodemographic characteristics from comparison and case groups.

	Comparison (*n* = 24)	Case (*n* = 60)	*p* value
Sociodemographic characteristics			
Age (years) (mean ± SD)	56.7 ± 11.6	62.7 ± 14.2	**0.0492** ^a^
Sex (female), *n* (%)	19 (79.2)	39 (65.0)	0.2045^b^
Smoking, *n* (%)	3 (12.5)	8 (13.3)	0.6645^c^
Alcoholism, *n* (%)	3 (12.5)	9 (15.0)	0.7307^c^
Use of sunscreen, *n* (%)	6 (25.0)	41 (68.3)	**0.0003** ^b^
Mean daily sunlight exposure (min) (mean ± SD)	35.0 ± 28.9	180.5 ± 123.7	**<0.0001** ^a^
Family history of cancer, *n* (%)	4 (16.7)	42 (70.0)	**<0.0001** ^b^
Race, *n* (%)			0.1377^b^
Caucasian	8 (33.3)	11 (18.3)	
Non-Caucasian	16 (66.7)	49 (81.7)	
Anthropometric parameters (mean ± SD)			
BMI (kg/m^2^)	27.3 ± 3.5	26.5 ± 3.7	0.2710^a^
TSF (mm)	20.2 ± 6.8	18.3 ± 7.8	0.1750^a^
MUAC (cm)	30.9 ± 3.1	30.1 ± 4.5	0.1910^a^
MAMC (cm)	24.6 ± 3.0	24.5 ± 4.0	0.7100^a^
Daily intake levels (mean ± SD)			
Vitamin A (*μ*g)	380.9 ± 197.1	558.0 ± 570.0	**0.0015** ^a^
Vitamin C (mg)	162.4 ± 419.3	57.9 ± 50.6	0.2223^a^
Vitamin E (mg)	6.1 ± 14.2	9.6 ± 7.0	**0.0018** ^a^
Cuprum (*μ*g)	1040.0 ± 1009.0	800.0 ± 400.0	**0.0331** ^a^
Zinc (*μ*g)	10.1 ± 12.6	6.5 ± 3.6	**0.0317** ^a^
Selenium (*μ*g)	52.2 ± 44.5	76.2 ± 38.8	**0.0285** ^a^

*N* = total of subjects/patients, SD = standard deviation, BMI = body mass index, TSF = triceps skinfold, MUAC = mid-upper-arm circumference, and MAMC = mid-arm muscle circumference. Statistical analysis: ^a^Mann-Whitney test; ^b^Chi-square test; ^c^Fisher's exact test.

**Table 3:** Comparison regarding sociodemographic characteristics from placebo and supplemented groups before supplementation.

	Placebo (*n* = 34)	Supplemented (*n* = 26)	*p* value
Sociodemographic characteristics			
Age (years) (mean ± SD)	65.6 ± 13.2	59.0 ± 14.9	0.0860^a^
Sex (female), *n* (%)	24 (70.6)	15 (57.7)	0.2994^b^
Smoking, *n* (%)	4 (11.8)	4 (15.4)	0.7172^c^
Alcoholism, *n* (%)	2 (5.9)	7 (26.9)	**0.0323** ^c^
Use of sunscreen, *n* (% )	22 (64.7)	19 (73.1)	0.4987^b^
Mean daily sunlight exposure (mean ± SD)	199.4 ± 130.2	155.8 ± 112.5	0.2450^a^
Family history of cancer, *n* (%)	24 (70.6)	18 (69.2)	0.9095^b^
Race, *n* (%)			0.5072^c^
Caucasian	5 (14.7)	6 (23.1)	
Non-Caucasian	29 (85.3)	20 (76.9)	
Anthropometric parameters (mean ± SD)			
BMI (kg/m^2^)	27.0 ± 4.1	25.8 ± 3.2	0.3440^a^
TSF (mm)	18.1 ± 7.4	17.5 ± 8.3	0.9100^a^
MUAC (cm)	30.0 ± 4.7	30.2 ± 4.2	0.6710^a^
MAMC (cm)	24.8 ± 4.0	24.1 ± 4.1	0.7670^a^
Daily intake levels (mean ± SD)			
Vitamin A (*μ*g)	705.3 ± 778.6	428.2 ± 232.4	0.8817^a^
Vitamin C (mg)	55.7 ± 47.6	54.2 ± 46.5	0.7326^a^
Vitamin E (mg)	9.0 ± 4.0	9.9 ± 9.3	0.6157^a^
Cuprum (*μ*g)	950.0 ± 510.0	870.0 ± 280.0	0.0784^a^
Zinc (*μ*g)	7.1 ± 4.4	5.8 ± 2.4	0.1321^a^
Selenium (*μ*g)	79.8 ± 47.4	76.9 ± 21.5	0.1979^a^

*N* = total of subjects/patients, SD = standard deviation, BMI = body mass index, TSF = triceps skinfold, MUAC = mid-upper-arm circumference, and MAMC = mid-arm muscle circumference. Statistical analysis: ^a^Mann-Whitney test; ^b^Chi-square test; ^c^Fisher's exact test.
